# Blood urea nitrogen to creatinine ratio is associated with in-hospital mortality among critically ill patients with cardiogenic shock

**DOI:** 10.1186/s12872-022-02692-9

**Published:** 2022-06-08

**Authors:** Di Sun, Changmin Wei, Zhen Li

**Affiliations:** Department of Cardiology, Shengli Oilfield Central Hospital, 31 Jinan Road, Dong ying, Shandong, China

**Keywords:** Blood urea nitrogen (BUN), Creatinine (Cr), BUN-to-Cr ratio (BCR), Cardiogenic shock, In-hospital mortality

## Abstract

**Backgrounds:**

Although Blood urea nitrogen (BUN) and serum creatinine concentration (Cr) has been widely measured in daily clinical practice, BUN-to-Cr ratio (BCR) for prognosis among patients admitted with cardiogenic shock (CS) remains unknown. The present study was conducted to assess the prognostic effectiveness of BCR on CS.

**Methods and results:**

Records of data for patients with CS were extracted from public database of the Medical Information Mart for Intensive Care-III (MIMIC-III). The primarily endpoint was in-hospital mortality. We incorporated multivariate Cox regression model and Kaplan–Meier curve to evaluate the relationship between BCR and in-hospital mortality, adjusting for potential confounders. Data of 1137 patients with CS were employed for the final cohort, with 556 in the low BCR (< 20) and 581 in the high BCR (≥ 20) group. In the multivariate Cox model and Kaplan–Meier curve, compared to low BCR, we found high BCR was independently associated with significantly improved in-hospital survival for CS (HR 0.66, 95% CI 0.51–0.84; *P* < 0.01). The benefit of high BCR on in-hospital survival for CS was remaining among subgroups of acute kidney injury (AKI) and non-AKI.

**Conclusions:**

Our analysis indicated that high BCR, as compared to low BCR, was correlated with improved in-hospital survival for participants with CS, with or without AKI. The results need to be proved in large prospective studies.

## Introduction

The definition of cardiogenic shock (CS) is characterized as inadequate forward tissue perfusion and excessive backward congestion due to cardiac pump insufficiency [1, 2]. Despite recent advancements in devices and managements in critical care unit for CS, in-hospital mortality rate remains high [3, 4]. The prevalence of CS in the ICU/ICCU dataset is 14–16% [5]. In-hospital mortality rates range from 30 to 60%, with nearly half of in-hospital deaths occurring within 24 h of onset, 70–80% of deaths occur 30–60 days after CS onset, indicating that the risk of death is time-dependent and clustered early after onset [6]. Therefore, early identification of high-risk CS patients is essential.

Blood urea nitrogen (BUN) and serum creatinine concentration (Cr) are both undergoing glomerular filtration and clinically used as important acute kidney injury (AKI) biomarkers [7]. Activation of neurohormonal axis, including sympathetic-nervous and renin–angiotensin–aldosterone system, as well as vasopressin, brings in the greatly reabsorption of BUN, while Cr cannot be reabsorbed [8–10]. Thus, elevated BUN to Cr ratio (BCR) is usually considered as a biomarker of neurohormonal activity. Activation of the neurohormonal system can exacerbate shock [11]. Indeed, CS commonly occurs after massive myocardial infarction or severe myocardial ischemia, resulting in impaired left ventricular function and decreased cardiac output and arterial blood pressure. Coronary perfusion is reduced, further impairing coronary reserve. Activation of the compensatory neurohormonal system, resulting in systemic vasoconstriction, tachycardia, and fluid retention. These above mechanisms exacerbate myocardial ischemia and produce a vicious cycle of increased ischemia, worsening myocardial function, and increased shock [12].

Recently, several studies have found that the BCR was associated with prognosis in patients with cardiac dysfunction [13–15]. However, the relationship between BCR and in-hospital mortality among CS was still unclarified, and we conducted this retrospective study to investigate this association.

## Methods

### Source of data

Record of data were extracted from the database of Medical Information Mart for Intensive Care-III (MIMIC-III), with an version 1.4, which contains comprehensive information for > 60,000 critically ill patients admitted to Beth Israel Deaconess Medical Center in USA between 2001 and 2012, and is freely available [16]. Approval for the usage of the database has already got from the Institutional Review Boards of Beth Israel Deaconess Medical Center and the Massachusetts Institute of Technology, thus it was no more needed by Institutional Review Boards in our institution.

### Study subjects

Patients identified as CS was selected, with the criteria for CS including international classification of disease-9 (ICD-9) codes were 785.51 or 998.01, and/or minimum systolic blood pressure lower 90 mmHg, or minimum systolic pressure higher than 90 mmHg with vasopressors support. Of these patients, age more than 18 years at admission was included. First admission was included, if one person who had multiple admissions, for the final analysis. Meanwhile, participants were excluded if (1) with more than 10% missing data; (2) values of the data exceeded the mean ± 3 times the standard deviation.

### Data extraction

We extracted the parameters of demographic, clinical, laboratory, and severity scoring from the database of MIMIC-III at admission in the first day: age, gender, ethnicity, type of admission (select, emergency, urgent); acute coronary syndrome (ACS) and AKI; BUN and Cr level; Simplified-Acute-Physiology-Score II (SAPSII); Acute-Physiology-Score III (APS III); the mean of heart rate, the mean of mean blood pressure; patients with supported by mechanical circulatory support (MCS), mechanical ventilation, as well as renal replacement therapy (RRT); patients using inotropes (milrinone and/or dobutamine), vasopressors (which incorporating norepinephrine, dopamine, phenylephrine, epinephrine), vasopressin,; patients with history of chronic heart failure, cardiac arrhythmias, cardiac valvular disease, hypertension, diabetes, chronic pulmonary disease, pulmonary circulation disease, renal disease, liver disease.

We defined the primary outcome as the in-hospital mortality, which was the time interval of patient survival at admission to hospital discharge.

### Statistical analysis

According to common clinical practice, participants in the present study was classified into low BCR (< 20) and high BCR (≥ 20) groups. Categorical data were shown as frequencies and percentages. Categorical data were compared between low BCR and high BCR groups by Chi-square or Fisher’s exact test. Variance analysis or the Wilcoxon test were used for statistical analysis of differences of continuous data in low BCR and high BCR groups.

Hazard ratio (HR) and 95% confidence interval (CI) for the relationship between BCR and in-hospital mortality were estimated with multivariate Cox regression model. Survival curve was expressed as Kaplan–Meier curve. The covariates were determined as potential confounders if they changed the estimates of BCR on the in-hospital mortality by more than 10% or were significantly related with in-hospital mortality, as well as on clinical judgement. Subgroup analysis were conducted among patients with or without AKI. The following covariates were selected on the basis of established associations. The following covariates were selected: age, gender, ethnicity, admission type, ASPSII, APSIII, MCS, RRT, inotropes and vasopressors, vasopressin, mechanical ventilation, mean blood pressure, mean heart rate, history of chronic heart failure, cardiac arrhythmias, pulmonary circulation disease, hypertension, diabetes, renal disease, liver disease.

We conducted statistical analyses by using R software (version 3.42), with a two sided *P* value < 0.05 was identified as statistically significant.

## Results

### Participant characteristics

**Fig. 1:**
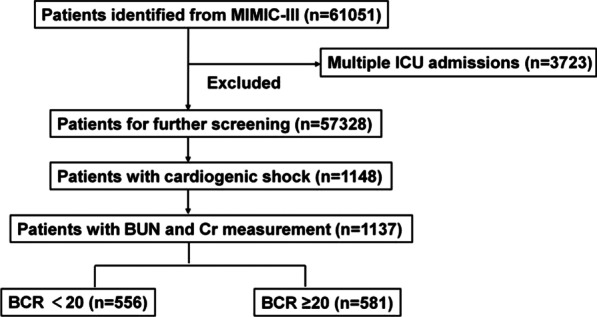
Flowchart of Patient Selection

Flowchart of patient selection was illustrated in Fig. 1 and 1137 critically ill patients with CS were enrolled for the final cohort analysis. The overall mean BCR was 21.58 ± 9.22. In the Table 1, characteristics of baseline for low and high BCR groups were demonstrated. Participants with high BCR were more likely to be elderly, female, and white and to report a history of cardiac arrhythmias, less hypertension, renal disease, and were less likely to use vasopressin and ventilator than those with low BCR.

**Table 1:** Baseline characteristics

	BUN-to-Cr ratio (BCR)		
Clinical parameters, *n* (%)	< 20 (n = 556)	≥ 20 (n = 581)	
Creatinine (Cr), mg/dL	2.25 ± 2.01	1.62 ± 0.94	< 0.01
Blood Urea Nitrogen (BUN), mg/dL	29.53 ± 19.92	44.69 ± 26.37	< 0.01
BUN-to-Cr ratio (BCR)	14.70 ± 3.75	28.16 ± 8.01	< 0.01
Age, years	67.72 ± 14.64	74.39 ± 11.96	< 0.01
Gender, *n* (%)			< 0.01
Female	199 (35.79%)	269 (47.33%)	
Male	357 (64.21%)	306 (52.67%)	
Ethnicity, *n* (%)			< 0.01
White	366 (65.83%)	410(70.57%)	
Black	51 (9.17%)	26 (4.48%)	
Other	139 (25.00%)	145(24.96%)	
Admission type			0.21
Select	43 (7.73%)	30 (5.16%)	
Emergency	492 (88.49%)	527 (90.71%)	
Urgent	21 (3.78%)	24 (4.13%)	
Mean Heart Rate, beats/min	90.21 ± 17.65	88.49 ± 16.28	0.09
Mean Blood Pressure, mmHg	73.66 ± 10.11	72.69 ± 9.73	0.10
APSIII	58.49 ± 24.42	58.14 ± 22.05	0.80
SAPSII	46.85 ± 15.87	48.39 ± 14.89	0.09
Inotropes, *n*(%)	215 (38.67%)	234 (40.28%)	0.58
Vasopressors, *n*(%)	454 (81.65%)	473(81.41%)	0.92
Dopamine, *n*(%)	240 (43.17%)	284 (48.88%)	0.053
Epinephrine, *n*(%)	116 (20.86%)	84 (14.46%)	< 0.01
Norepinephrine, *n*(%)	344 (61.87%)	323 (55.59%)	0.03
Phenylephrine, *n*(%)	209 (37.59%)	214 (36.83%)	0.79
Vasopressin, *n*(%)	161 (28.96%)	135 (23.24%)	0.03
MCS, *n*(%)	216 (38.85%)	196 (33.73%)	0.07
RRT, *n*(%)	72 (12.95%)	12 (2.07%)	< 0.01
ACS, *n*(%)	283 (50.90%)	305 (52.50%)	0.59
Chronic Heart Failure, *n*(%)	112 (20.14%)	138 (23.75%)	0.14
Cardiac Arrhythmias, *n*(%)	87 (15.65%)	119 (20.48%)	0.03
Valvular Heart Disease, *n*(%)	48 (8.63%)	48 (8.26%)	0.82
Hypertension, *n*(%)	118 (21.22%)	94 (16.18%)	0.03
Diabestes, *n*(%)	182 (32.73%)	216 (37.18%)	0.12
Chronic Pulmonary Disease, *n*(%)	95 (17.09%)	114 (19.62%)	0.27
Renal Disease, *n*(%)	151 (27.16%)	116 (19.97%)	< 0.01
Liver Disease, *n*(%)	23 (4.14%)	18 (3.10%)	0.35
Length of Hospital Stay, days	12.25 ± 11.50	12.84 ± 11.32	0.38
In-hospital Deaths, *n*(%)	230 (41.37%)	228 (39.24%)	0.47

*Plus–minus values are means ± SD. APSIII Acute Physiology Score III, SAPSII Simplified Acute Physiology Score II, MCS mechanical circulatory support, RRT renal replacement therapy

### Relationship between BCR and in-hospital mortality

**Fig. 2:**
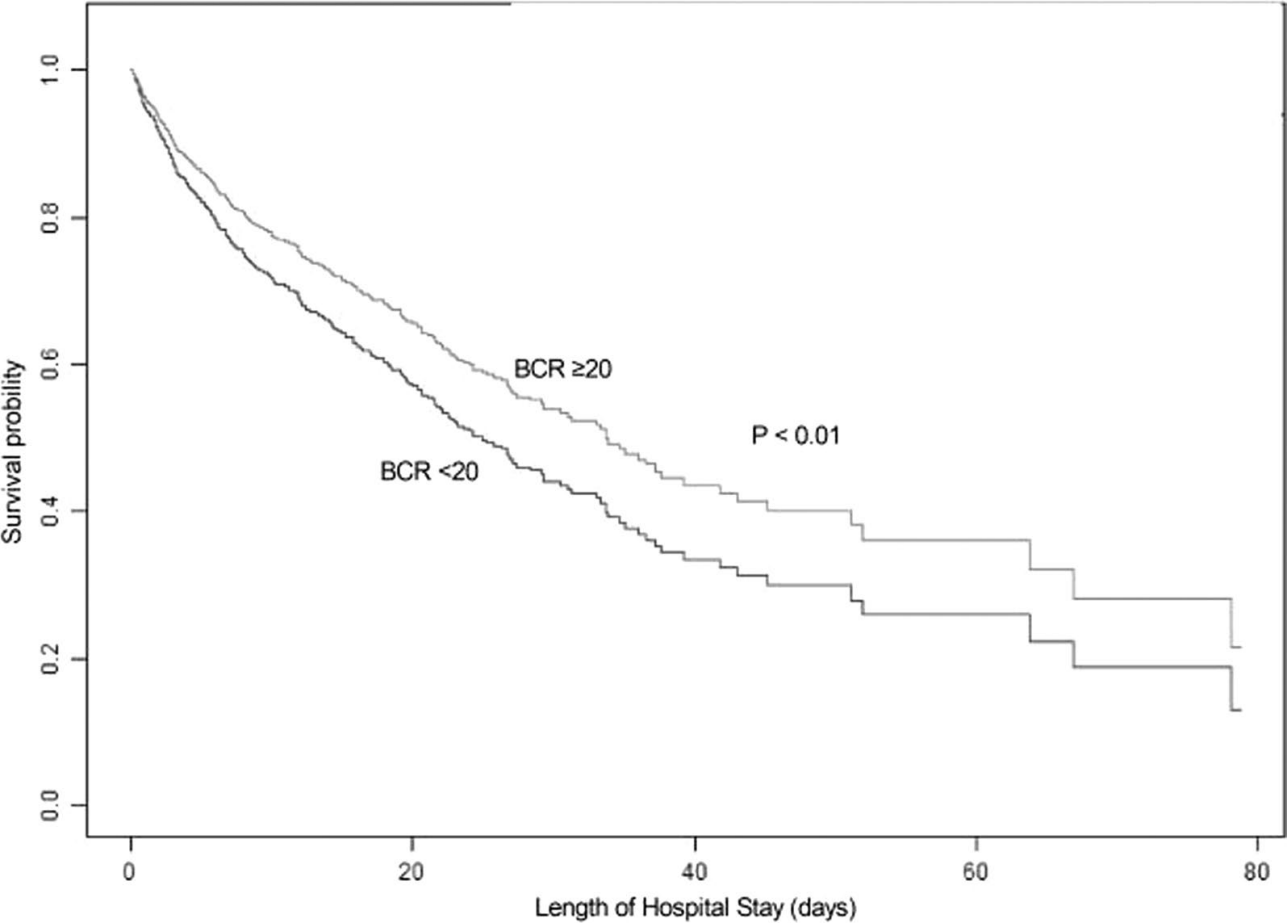
Kaplan–Meier Survival from in-hospital mortality for patients in BCR < 20 and BCR ≥ 20

A total of 458 in-hospital deaths occurred during the follow-up period. Figure 2 was the Kaplan–Meier curve estimate. Multivariate Cox model results demonstrated that higher level of BCR was related with decreased risk of in-hospital mortality with HR of 0.66 (95% CI 0.51–0.84; *P* < 0.01) in participants with CS, after adjustment of age, gender, ethnicity, type of admission, ASPSII, APSIII, MCS, RRT, inotropes, vasopressors, vasopressin, mechanical ventilation, mean blood pressure, mean heart rate, chronic heart failure, cardiac arrhythmias, pulmonary circulation disease, hypertension, diabetes, renal disease, liver disease, as shown in Table 2. The calibratation using Hosmer–Lemeshow statistics was 0.6862.

**Table 2:** Association between BCR and in-hospital mortality by Cox regression after multivariable model

	Death, *n*(%)	HR (95% CI)	*P* value
Multivariable model			
Low BCR	230 (41.37%)	1	
High BCR	228 (39.24%)	0.66 (0.51, 0.84)	< 0.01

HR, Hazard Ratio; CI, confidence interval. Multivariable model, adjusted for age, gender, ethnicity, admission type, APSIII, ASPSII, using MCS, mechanical ventilation, RRT, inotropes, vasopressors, vasopressin, mean heart rate, mean blood pressure, and history of chronic heart failure, cardiac arrhythmias, pulmonary circulation disease, hypertension, diabetes, renal disease, liver disease

### Subgroup analysis for patients with or without AKI

**Fig. 3:**
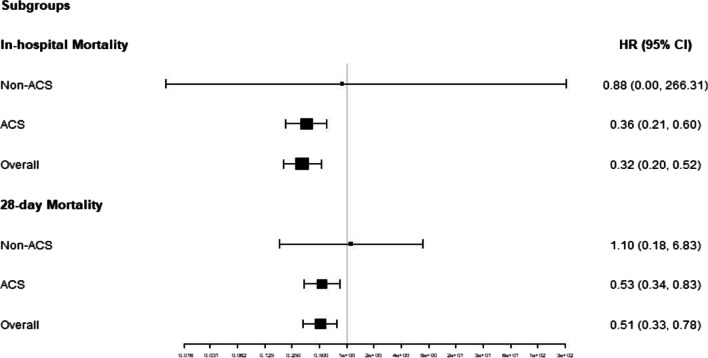
Subgroup analyses for HR (BCR ≥ 20 to BCR < 20) among patients with AKI of 0.72 (0.53,0.97) and non-AKI of 0.66 (0.51,0.84)

Among patients with AKI and non-AKI, subgroup analysis for the association between BCR and in-hospital mortality, were remaining consistent, with AKI of HR 0.72, (95% CI 0.53–0.97; *P* = 0.03), and non-AKI of HR 0.57 (95% CI 0.35–0.95; P = 0.03), as shown in Fig. 3.

## Discussion

In the present retrospective study, we found that a high level of BCR (≥ 20) was associated with improved risk of in-hospital survival, with HR of 0.66, after adjusting for the confounders, including BUN and Cr. This relationship was still statistically significant in CS patients among AKI and non-AKI subgroups.

Previous studies also have found several indexes with prognostic predictive value among patients with CS. Çinar et al. [17] has demonstrated a prognostic value of creatinine in patients with ST-elevation myocardial infarction-related cardiogenic shock. Another study [18] found GRACE score was an independent prognostic marker of AKI in patients with CS complicated with STEMI. The BUN and Cr, even both filtered in the kidney, only BUN not Cr is reabsorbed after filtration. Indeed, BUN reabsorption is greatly influenced by the activation of neurohormonal system, which including system of sympathetic-nervous, renin–angiotensin–aldosterone, as well as vasopressin [19, 20]. Therefore, the BCR, a combination of BUN to Cr ratio, is usually served as a biomarker of neurohormonal activity that was shown to relate the prognostic endpoints of cardiac dysfunction [13–15]. In one large cohort study of acute heart failure, Matsue et al. reported that the higher values of BCR were independently predictive of worse outcome and provided additional prognostic value on previous risk factors, including Cr and BUN [11]. Another retrospective study found that BCR ≥ 20 among patients with congestive heart failure complicated with AKI was strongly correlated with death [12]. Murata et al. also demonstrated that patients in heart failure with BCR ≥ 20.3 had a lower survival probability, even after adjusting of Cr and BUN [15] To our knowledge, the prognostic value of BCR on admission has not been reported in critically ill patients with cardiogenic shock, which was the most severe form state of acute heart failure. In the current study, we found that BCR ≥ 20 was associated with lower in-hospital mortality, as compared to low BCR, after adjustment for important potential covariates, including BUN and Cr. Subgroup analysis showed that the protective effective of high BCR was consistent among patients with or without AKI.

Compared to association of the poor outcome and high BCR in patients with acute heart failure, the severer form of CS populations in our study might explain for the robust prognosis of high BCR. Hypoperfusion of vital organs is a hallmark of CS, and the decrease in cardiac output triggers the release neurohormone of catecholamines, angiotensin, and vasopressin, which may lead to improvement in organ perfusion. Indeed, in current clinical practice, catecholamine vasoconstrictors are still the mainstay therapies for the early stage of cardiogenic shock [21–23], as well as vasopressin and angiotensin are both indicated for management of shock patients [24, 25].

Limitations of our study should also be noted. First of all, due the nature of retrospective observational study, we could not prove causality. Second, the BCR was extracted only on admission and in a single center (Beth Israel Deaconess Medical Center), with selection bias should not be ignored. Third, we could not include some essential data in the final analysis due to excess missing (more than 20% missing) such as lactate, brain natriuretic peptide, crp, as well as left ejection fraction, which may be relevant to mortality. Fourth, BCR may have been altered with drugs on the patients previous had taken, which were also missing in our study.

## Conclusions

In conclusion, a higher level BCR at admission, was independently correlated with lower in-hospital mortality in participants with CS. Meanwhile, this robust outcome of in-hospital survival for high BCR group was consistent among participants with or without AKI. These findings need to be prospective confirmed by large prospective trial.

## Data Availability

The datasets generated and/or analysed during the current study are available in the [MIMIC-III database] repository, [https://mimic.mit.edu/].

## Abbreviations

BUN: Blood urea nitrogen
Cr: Creatinine
BCR: BUN-to-Cr ratio
CS: Cardiogenic shock
MIMIC-III: Medical Information Mart for Intensive Care-III
AKI: Acute kidney injury
ACS: Acute coronary syndrome
SAPSII: Simplified-Acute-Physiology-Score II
APS III: Acute-Physiology-Score III
MCS: Mechanical circulatory support
RRT: Renal replacement therapy
HR: Hazard ratio
CI: Confidence interval
